# Eye nurse training in the Pacific islands

**Published:** 2020-12-31

**Authors:** Pawan Baral

**Affiliations:** 1 Research Officer: Himalaya Eye Hospital, Pokhara, Nepal.

**A training programme in Fiji and Papua New Guinea is rapidly increasing the number of ophthalmic nurses able to support eye care delivery in the challenging Pacific Island Countries region.**

**Figure:**
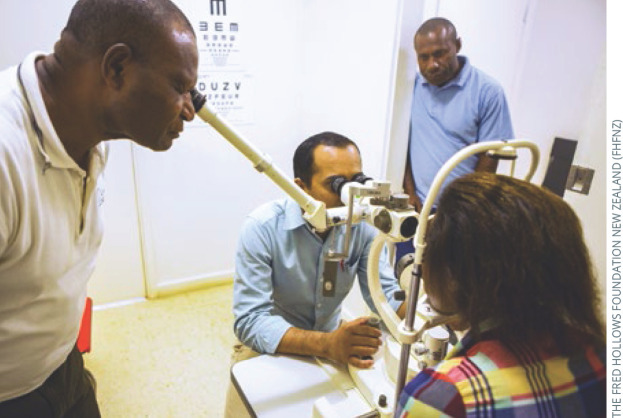
An ophthalmic nurse learns to examine eyes through a slit lamp during their ophthalmic nurse diploma course. **PAPUA NEW GUINEA**

Papua New Guinea and Fiji are two of the 25 nations and territories that make up the Pacific Island Countries.

Until 2006, there were almost no ophthalmic nurses, and very few ophthalmologists and optometrists, in the region. To respond to this challenge, the Fred Hollows Foundation New Zealand (FHFNZ) established the Pacific Eye Institute in Fiji in 2006 and a similar training programme in Papua New Guinea in 2007, and began enrolling 10-15 nurses per year in the Postgraduate Diploma in Eye Care. This one-year, 120-credit programme is offered in Fiji by the Fiji National University and in Papua New Guinea by the Divine Word University. It is accredited by the International Joint Commission on Allied Health Personnel in Ophthalmology (JCAHPO).

In Fiji, the entry requirements include a nursing qualification, a minimum of one year of work experience (post-internship, in a health-related field), and observation in an eye clinic for a minimum of one month to ensure the candidate has the interest and motivation to pursue a career in eye care. Graduates are registered by the Fiji Nursing Council as eye nurse specialists.

**The role of professional associations**

The ophthalmic clinicians in Papua New Guinea established a professional association named the Ophthalmic Clinicians Association within the National Department of Health. All ophthalmic clinicians in Papua New Guinea can, by default, be a member of the Ophthalmic Clinicians Association. The other Pacific Island Countries have a larger forum named the Pacific Eye Care Society (PacEYES). PacEYES is part of the International Council of Ophthalmology and includes other eye health professionals. By paying an annual membership fee, all eye care practitioners in Papua New Guinea and the Pacific islands can be members of PacEYES.

**Figure:**
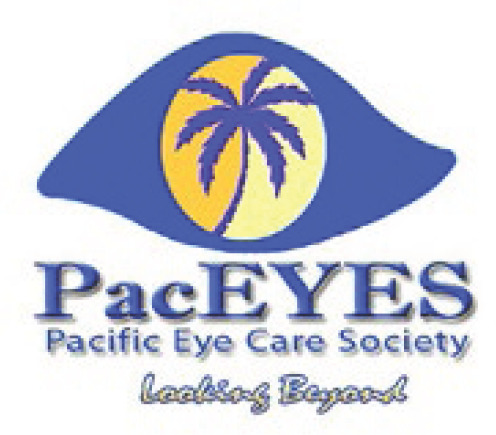

Through these associations, the eye nurses in the region organise national and regional conferences, advocate, and plan eye care activities. This has enabled ophthalmic nurses in the region to share best practice and forge collaborative clinical and research partnerships and networks, both regionally and internationally. PacEYES has the potential to develop as a college for upskilling all levels of eye care practitioners in the region.

For information on how to join PacEYES, visit **www.paceyes.org/home**

In Papua New Guinea, candidates must have a Bachelor's degree in a health sciences field such as nursing, health extension, or medicine. However, applicants with a three-year diploma in a health science may apply for recognition of prior learning if they can demonstrate that they have workplace experience equivalent to the final year of a Bachelor's degree in a health sciences programme. The course is accredited by the Department of Higher Education, Research Science and Technology, and graduates are registered by the Papua New Guinea medical board as ‘ophthalmic clinicians.’ This grants them the right to prescribe Category C topical ophthalmic drugs for eye disease treatment.

Candidates undergo training in the diagnosis and management of common eye conditions, refraction and spectacle prescription, ophthalmic operating theatre procedures, community eye care, eye health promotion, and diabetic retinopathy screening using indirect biomicroscopy and fundus photography. Depending on the scope of practice in different countries, the level of eye care provided by the ophthalmic nurses may vary from diagnosis and referral to diagnosis and treatment.

More than 200 ophthalmic nurses have been trained to date. The original target of 241 ophthalmic nurses (which meets the World Health Organization's target ratio for the region, of 1 ophthalmic nurse per 50,000 population), was increased to 300 to compensate for future attrition, population growth, and the geographical spread of the population.

The Postgraduate Diploma in Eye Care offers graduates ongoing training and further education as well as support in their new place of work,^1^ which helps to retain them in the workforce and ensure they are able to do what they were trained to do.

In just over a decade, eye care in the Pacific Island Countries has grown from a handful of eye practitioners and eye care services to a significant number of eye practitioners providing eye care services through local ministries of health. Graduates are also now leading the training in both Fiji and Papua New Guinea.
